# Extracellular Bacterial Pathogen Induces Host Cell Surface Reorganization to Resist Shear Stress

**DOI:** 10.1371/journal.ppat.1000314

**Published:** 2009-02-27

**Authors:** Guillain Mikaty, Magali Soyer, Emilie Mairey, Nelly Henry, Dave Dyer, Katrina T. Forest, Philippe Morand, Stéphanie Guadagnini, Marie Christine Prévost, Xavier Nassif, Guillaume Duménil

**Affiliations:** 1 INSERM, U570, Paris, France; 2 Université Paris Descartes, Faculté de Médecine Paris Descartes, UMR S570, Paris, France; 3 CNRS, UMR 168, Paris, France; 4 University of Wisconsin-Madison, Department of Bacteriology, Madison, Wisconsin, United States of America; 5 Plate-Forme de Microscopie Electronique, Institut Pasteur, Paris, France; 6 AP-HP, Hôpital Necker-Enfants Malades, Paris, France; Northwestern University Feinberg School of Medicine, United States of America

## Abstract

Bacterial infections targeting the bloodstream lead to a wide array of devastating diseases such as septic shock and meningitis. To study this crucial type of infection, its specific environment needs to be taken into account, in particular the mechanical forces generated by the blood flow. In a previous study using *Neisseria meningitidis* as a model, we observed that bacterial microcolonies forming on the endothelial cell surface in the vessel lumen are remarkably resistant to mechanical stress. The present study aims to identify the molecular basis of this resistance. *N. meningitidis* forms aggregates independently of host cells, yet we demonstrate here that cohesive forces involved in these bacterial aggregates are not sufficient to explain the stability of colonies on cell surfaces. Results imply that host cell attributes enhance microcolony cohesion. Microcolonies on the cell surface induce a cellular response consisting of numerous cellular protrusions similar to filopodia that come in close contact with all the bacteria in the microcolony. Consistent with a role of this cellular response, host cell lipid microdomain disruption simultaneously inhibited this response and rendered microcolonies sensitive to blood flow–generated drag forces. We then identified, by a genetic approach, the type IV pili component PilV as a triggering factor of plasma membrane reorganization, and consistently found that microcolonies formed by a *pilV* mutant are highly sensitive to shear stress. Our study shows that bacteria manipulate host cell functions to reorganize the host cell surface to form filopodia-like structures that enhance the cohesion of the microcolonies and therefore blood vessel colonization under the harsh conditions of the bloodstream.

## Author Summary

Certain infectious agents reach the bloodstream and succeed in surviving and multiplying at this site. This stage of the infection is associated with a life-threatening condition. The Gram-negative bacterium meningococcus, responsible for septicemia and meningitis, stands out as a paradigm of such a pathogen. Despite the characteristic flow-generated hydrodynamic forces of the bloodstream, meningococci have the striking ability to bind to the endothelium and to multiply in bacterial aggregates called microcolonies. Meningococci form aggregates in absence of eukaryotic cells, but we show that such aggregates are sensitive to mechanical stress, indicating that the presence of host cells enhances microcolony cohesion. Consistently, analysis of meningococcal microcolonies growing on the host cellular surface reveals that these structures are dense with cellular material in the form of cellular protrusions. Blocking this bacteria-induced cellular response renders microcolonies sensitive to blood flow. We then identify a bacterial component located on the outside of the bacteria and in direct contact with host cells as a key factor in the induction of this cellular response. This bacteria-induced cellular response is therefore a striking example of how pathogens exploit cellular functions as a survival strategy, in this case in the particular context of the bloodstream.

## Introduction

Infectious diseases leading to colonization of the blood by the infectious agent are a major burden to society. Such infections lead to devastating clinical manifestations including septic shock, hemorrhagic syndromes or infection of the brain (meningitis). Pathogens triggering such diseases are diverse and include viruses, bacteria (Gram-positive and Gram-negative), parasites and fungi. The common characteristic of these pathogens is their presence in the bloodstream at a given point of the infection process. Such pathogens are exposed to mechanical forces exerted by the blood flow, which follows a complex pattern throughout different blood vessels. This specific environment is increasingly recognized as a determining factor during pathogenesis and implies an adaptation of the pathogens [1]–[3]. One such infectious agent, used as a model in this study, is the Gram-negative bacteria *Neisseria meningitidis* [4].

*N. meningitidis* infection leads to two distinct clinical manifestations, a rapidly evolving form of septicemia or meningitis. The only known reservoir is the human nasopharynx, where the bacterium multiplies without causing symptoms in 10–20% of the human population who serve as carriers[5]. At a low frequency, bacteria cross the epithelial barrier and access the bloodstream, causing septicemia. In the bloodstream, *N. meningitidis* interact with endothelial cells, cross the blood brain barrier (BBB) and proliferate in the brain [4],[6].

One property of this pathogen thought to be key both in nasopharynx colonization and in disease development is its ability to adhere to host cells. As is the case for numerous pathogens, adhesive properties of *N. meningitidis* are mediated by filamentous organelles designated type IV pili [7]. In addition to adhesion, type IV pili allow bacteria to spontaneously form large aggregates in suspension and on the cellular surface. In *N. meningitidis*, expression of functional type IV pili on the bacterial surface requires 22 *pil* genes. The main and structural component of a pilus is the pilin, a protein encoded by the *pilE* gene in *N. meningitidis*. After being cleaved by the prepilin peptidase PilD, pilin subunits assemble in a helical fiber in the periplasm and exit the outer membrane through a pore [8]. In pathogenic *Neisseria* species, there are seven pilin-like proteins, so-called due to their conserved N-terminal PilD cleavage sequences: PilH, PilI, PilJ, PilK, ComP, PilV and PilX (referred to as PilL in *N. gonorrheae*) [9]. PilH, I, J and K are essential for Tfp biogenesis since the corresponding mutants are non-piliated [9],[10]. In contrast, *comP*, *pilV* and *pilX* mutants are piliated, so the corresponding proteins are thought to insert in the pilus fiber and mediate specific type IV pili dependent functions [9], [11]–[14].

We previously demonstrated the importance of mechanical forces generated by the bloodstream on the process of pathogenesis [4]. In the bloodstream, shear stress levels vary between 5 and 100 dynes/cm^2^ with values reaching 40 dynes/cm^2^ in capillary networks commonly colonized by *N. meningtidis* during infection [15]. Initial binding to host cells is strongly inhibited by shear stress and can only occur in capillaries where shear stress occasionally decreases to values below 0.5 dynes/cm^2^ for short periods of time before increasing back to values around 40 dynes/cm^2^. After initial binding, bacteria proliferate on the cell surface and form large microcolonies strikingly resistant to high external forces generated by the bloodstream. These microcolonies eventually fill the entire lumen of the capillaries and could strongly affect the barrier function of the endothelium. The ability of these large bacterial colonies to resist blood flow on the surface of endothelial cells in brain capillaries is therefore a probable determining factor in subsequent crossing of the BBB. The mechanical environment of microcolonies is also important during nasopharynx colonization. Bacterial microcolonies forming on the epithelium are exposed to high levels of shear stress due to mucus flow, sneezing and coughing.

Like several extracellular pathogens including Group A *Streptococci* [16] and *Bartonella spp.* [17], *N. meningitidis* triggers a local remodeling of the eukaryotic cell membrane. In the case of *N. meningitidis*, surface remodeling results in numerous cell membrane projections that can be found inside and around microcolonies adhering to the cell surface [6],[18],[19]. Induction of these cellular projections was proposed to be the initial step of an invasion process leading to crossing of cellular barriers by transcytosis [6]. However, the low efficiency of invasion (approx. one bacteria per thousand completes transcytosis) suggests that the potent bacteria-induced remodeling of the host cell surface has other functions.

*In vitro* analysis of infection in culture allowed description of this cellular response at the molecular level. A profound actin cytoskeleton reorganization was found to take place at the base of bacterial microcolonies [20] leading to the formation of a honeycomb lattice structure called “cortical plaque”. Ezrin, an actin binding protein member of the ERM (Ezrin/radixin/moesin) family, is abundantly recruited to the tip of cellular projections surrounding *N. meningitidis* [18]. Furthermore, in addition to ERM family proteins, numerous host cell transmembrane proteins such as ICAM-1, CD44, CD46 and ErbB2 are clustered under microcolonies [18], [20]–[22].

In the closely related pathogen *N. gonorrhoeae*, mutants defective in the biosynthesis of type IV pili were shown to be unable to induce host cell membrane remodeling [20]. The ability of type IV pili to retract is important for optimal signaling, but if there is a specific component of type IV pili beyond retraction force *per se* involved in triggering the cellular response, it remains to be identified [23].

The goal of this study was to identify the molecular mechanism allowing large bacterial aggregates to resist the high mechanical forces exerted by the blood flow in brain capillaries during pathogenesis and by mechanical forces operating in the nasopharynx during colonization. Hence, the study was conducted mostly on endothelial or in some cases epithelial cells. We first show that bacterial auto-aggregation is insufficient to explain the mechanical resistance of microcolonies growing on the host cell surface. Pharmacological compounds targeting specific cellular functions were then used to identify processes involved in increasing microcolony cohesion. Finally we identified bacterial factors involved in triggering the cellular response underlying the increased mechanical resistance of microcolonies.

## Results

### Type IV pili–dependent bacterial autoaggregative properties are insufficient to explain the mechanical resistance of microcolonies growing on the cellular surface

We previously showed that *N. meningitidis* growing in tight bacterial aggregates on eukaryotic cellular surfaces are highly resistant to external forces exerted by the harsh conditions found in the nasopharynx or in the bloodstream [4]. As this property of meningococci is likely to be a determining factor of nasopharynx colonization and pathogenesis we undertook to further explore its molecular basis. When proliferating in suspension, *N. meningitidis* form bacterial aggregates of different sizes, spontaneously and independently of the adhesion to host cells; this process depends on type IV pili. Our first hypothesis was therefore that bacteria-bacteria interactions could explain the mechanical resistance of bacterial colonies growing on the cellular surface. To test this we compared cohesive forces involved in bacterial aggregates growing on the cellular surface *vs.* forces for aggregates growing in suspension by studying their rupture under controlled shear stress.

**Figure 1:**
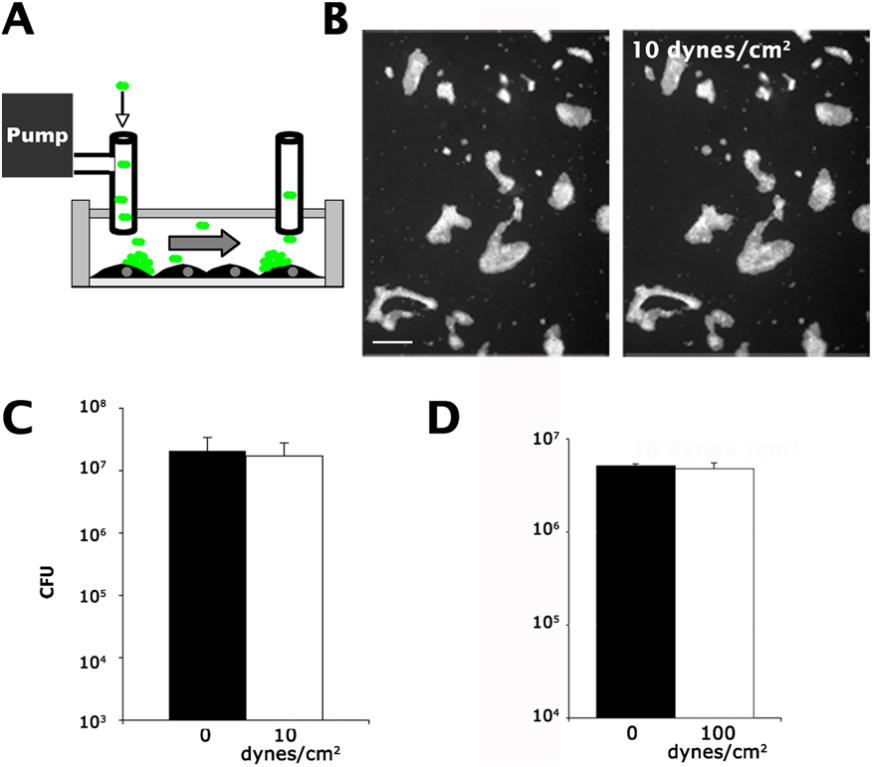
Bacterial aggregates adhering to host cells are highly resistant to shear stress. Bacterial aggregates on the cellular surface were submitted to shear stress and the ability of the aggregates to remain intact was compared (scale bars corresponds to 50 µm). (A) A monolayer of endothelial cells was infected with *N. meningitidis* expressing GFP for a period of 3 hours to allow microcolony formation as observed by fluorescence microscopy. Infected cells were then submitted to 10 dynes/cm^2^ in a laminar flow chamber as depicted on the diagram for a period of 5 min. (B) Aggregates remained unchanged before and after application of 10 dynes/cm^2^ (compare left and right panels). (C) The number of colony forming units before (black bars) and after (white bars) application of 10 dynes/cm^2^ was determined by a dilution plating and CFU determination. (D) The effect of 100 dynes/cm^2^ was determined.

Bacteria were exposed to shear stress in a laminar flow chamber (Figure 1A) as previously described [4]. GFP-expressing bacteria were allowed to grow on cells until they formed microcolonies, at which point mechanical force was introduced with liquid flow. As expected, shear stress could be increased to 10 dynes/cm^2^ for a period of 5 min without any sign of detachment (Figure 1B, compare left and right panels). To quantify the effect of flow, bacteria growing on cells were collected and plated to determine the number of colony forming units (CFU, Figure 1C). The number of CFUs did not change after the introduction of flow. Bacterial colonies were in fact resistant to shear stress levels of 100 dynes/cm^2^, a value which represents the highest forces operating in large blood vessels (Figure 1D). Under the assumption that bacteria behave as spherical beads, it can be estimated that a shear stress of 100 dynes/cm^2^ exerts a force of about 300 pN on a single bacterium, or 30 nN on a 10 µm wide colony [24]. When growing on cells, bacterial aggregates are therefore highly resistant to mechanical stress.

**Figure 2:**
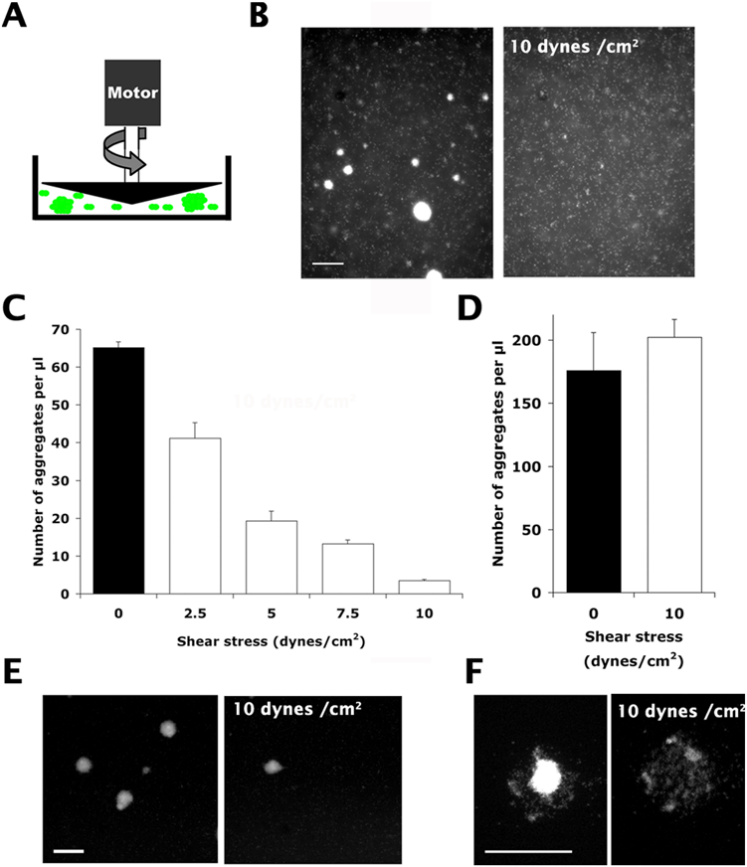
Bacterial aggregates in suspension are sensitive to shear stress. Bacterial aggregates in the absence of host cells were submitted to shear stress and the ability of the aggregates to remain intact was compared (scale bars corresponds to 50 µm). (A) GFP-expressing *N. meningitidis* proliferating in suspension in cell culture medium were analyzed under a microscope to visualize aggregates of whose number and size were determined by microscopy and automated image analysis. Bacterial aggregates were submitted to 2.5–10 dynes/cm^2^ shear stress levels in a cone and plate device as depicted on the diagram and aggregates analyzed. (B) Aggregates in suspension were disrupted after application of 10 dynes/cm^2^ (compare left and right panels). (C) The effect of different shear stress levels on the number of bacterial aggregates was determined. (D) The effect of shear stress was determined for the *pilT* strain deficient for pilus retraction. (E) Bacteria were immobilized on a glass slide coated with a monoclonal antibody directed against type IV pili, allowed to proliferate and colonies were submitted to 10 dynes/cm^2^. (F) Higher magnification view of a colony immobilized on a glass slide before and after shear stress application.

The resistance of bacterial aggregates growing in suspension and in the absence of cells was then tested using a cone and plate device to ensure a homogeneous shear stress throughout the entire sample (Figure 2A). Bacteria were grown in suspension under conditions allowing the formation of aggregates of various sizes, similar to those found on the surface of human cells (7–15 µm in diameter). Under the same conditions, a mutant lacking type IV pili did not form any aggregates (not shown). Introduction of 10 dynes/cm^2^ dramatically decreased the number of aggregates (Figure 2B, compare left and right panels). To quantify the effect, the size distribution of the aggregates was then determined microscopically by automatic image analysis. In absence of shear stress, aggregates ranged over a wide size distribution with the smallest aggregates involving only a few bacteria and others occasionally reaching sizes up to 10,000 bacteria. For further analysis we limited our analysis to a window of sizes between 500 and 5000 bacteria to match aggregate sizes found on the cell surface. Such suspensions of bacterial aggregates were submitted to different levels of shear stress varying between 2.5 and 10 dynes/cm^2^ and the number of aggregates was determined (Figure 2C). Shear stress levels as low as 2.5 dynes/cm^2^ were sufficient to significantly affect the number of aggregates in the suspension and the effect increased with the intensity of shear. Application of 10 dynes/cm^2^ led to a nearly twenty-fold decrease in the number of aggregates. As a comparison, the effect of shear stress on aggregates formed by the *pilT* mutant was determined. The *pilT* mutant is characterized by a defect in pilus retraction, bacteria are hyperpiliated and the *pilT* strain forms aggregates that are unusually large and numerous. Application of 10 dynes/cm^2^ shear stress had no effect on the number of aggregates (Figure 2D). Sensitivity of aggregates in suspension to shear stress is therefore a pilus retraction-dependent property.

To confirm these results under conditions more closely mimicking those found when bacteria are growing on the cellular surface in the flow chamber, bacteria were immobilized on a glass surface with an adsorbed antibody directed against the major pilin and allowed to proliferate before their mechanical resistance was evaluated. Bacteria growing on the glass surface formed large aggregates similar in shape and size to those found on cells. Introduction of 10 dynes/cm^2^ led to the progressive detachment of the colonies (Figure 2E, compare left and right panels). Higher magnification observation shows that a monolayer of bacteria remains attached to the glass slide after application of shear stress (Figure 2F). Taken together, these results show that wild type *N. meningitidis* bacterial aggregates are sensitive to shear stress levels in the range of 2.5–10 dynes/cm^2^ when not attached to host cells. This indicates that the forces engaged in the cohesion of the suspended aggregates were at least one order of magnitude lower than those ensuring mechanical resistance of microcolonies formed by *N. meningitidis* at the surface of host cells.

### Lipid microdomain disruption by cholesterol depletion of the host cell plasma membrane renders microcolonies sensitive to mechanical stress

The sensitivity to shear stress of bacterial aggregates in suspension suggests that host cells play an active role in conferring mechanical resistance to bacterial microcolonies on the cell surface. To identify the cellular components involved in conferring mechanical resistance to microcolonies, inhibitors of several cellular processes were tested. Results presented here focus on drugs targeting the actin cytoskeleton (cytochalasin D), the microtubule network (nocodazole) and plasma membrane lipid composition (methyl-ß-cyclodextrin (MßCD)).

**Figure 3:**
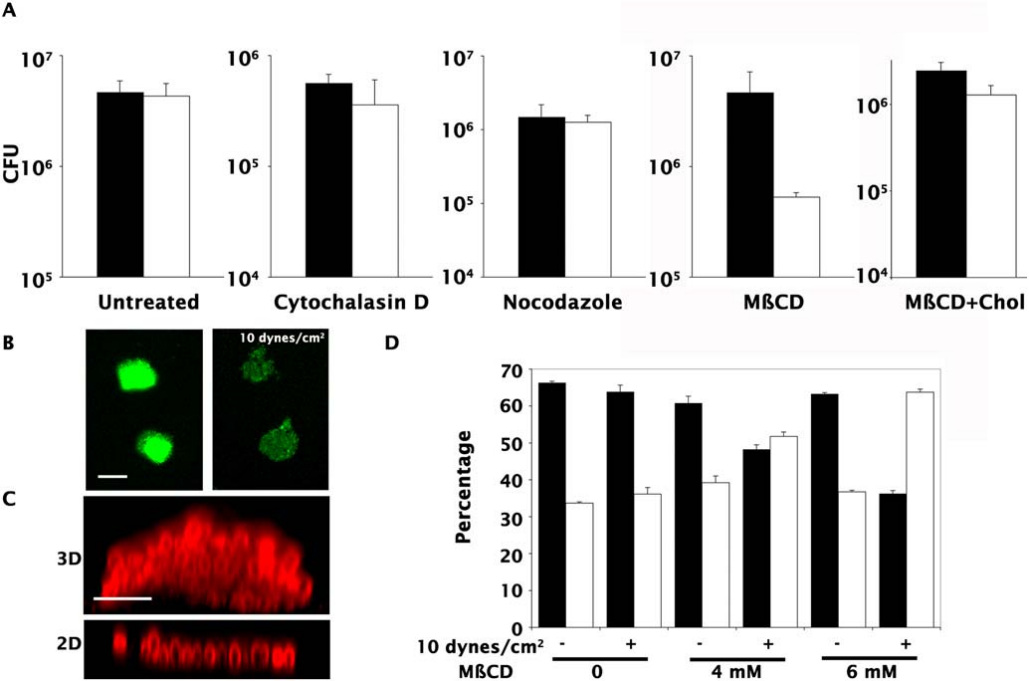
Host cell cholesterol depletion with cyclodextrin renders bacterial microcolonies sensitive to shear stress. Implication of specific cellular functions was tested using inhibitors targeting actin cytoskeleton, microtubules or plasma membrane cholesterol. (A) The effect of increasing shear stress on GFP-expressing wild type microcolonies growing on cells treated with cytochalasin D, Nocodazole, or methyl ß cyclodextrin (MßCD) was analyzed by a plating assay, before (black bars) and after flow increase (10 dynes/cm^2^, white bars). The effect of cholesterol repletion is also indicated (MßCD+Chol). (B) Application of shear stress on infected cells treated with MßCD led to the appearance of numerous flat two-dimensional microcolonies (right panel) visible under the fluorescence microscope in contrast with the large three-dimensional microcolonies (left panel) observed on untreated cells (scale bar correspond to 10 µm). (C) Images of 2D and 3D microcolonies were taken with a confocal microscope and the Z-section is presented (scale bars correspond to 3 µm). (D) The frequency of 2D (white bars) and 3D (black bars) microcolonies was determined under the different conditions and expressed as the percentage of the total number of colonies.

Cytochalasin D (1 µM) and nocodazole (1 µM) treatment did not affect the cohesion of microcolonies, indicating that neither the actin nor microtubule cytoskeleton was involved in conferring mechanical resistance (Figure 3A, Cytochalasin D and Nocodazole). The efficiency of these drugs was monitored by immunofluorescence visualization of their effects on F-actin and microtubules. In contrast, treatment with MßCD at a concentration of 4 mM led to a twenty-fold decrease in the number of adherent bacteria (4.7×10^7^ *vs.* 2.5×10^6^). By decreasing the cholesterol concentration in the plasma membrane, MßCD inhibits the formation of lipid rafts, a process necessary for numerous cellular signaling pathways [25]. Among our panel of inhibitors, MßCD was the only drug that had an effect on the mechanical resistance of microcolonies. The effect was reversed by repletion of cholesterol (Figure 3A, MßCD+Chol). Video microscopy revealed that after MßCD addition and flow increase, microcolonies were less tightly associated with the cellular surface and found to be progressively disrupted, leaving a monolayer of bacteria directly in contact with the cellular surface (Figures 3B, Video S1 and S2). Importantly, MßCD did not affect the ability of the bacteria to form aggregates in suspension nor did it affect the amount of pili present on bacteria growing in suspension or on cells as seen by immunofluorescence (data not shown). The two- vs. three-dimensionality of microcolonies was easily discernible by phase contrast microscopy but can be best documented with confocal microscopy (Figure 3C). The frequency of two- and three-dimensional microcolonies was determined following MßCD treatment and the application of shear stress (Figure 3D, white and black bars respectively). In the absence of shear stress, 66+/−0.3% of microcolonies were multilayered. As expected, in the absence of the drug, microcolonies were resistant to shear stress. Upon treatment of cells with 6 mM MßCD, the frequency of three-dimensional colonies dropped from 63+/−0.4% to 36+/−0.8% following application of flow. The effect was concentration dependent, with intermediate values at a concentration of 4 mM MßCD.

Strikingly, disruption of the actin and microtubule cytoskeletons did not affect microcolony cohesion. In contrast, altering plasma membrane composition by depleting plasma membrane cholesterol pools rendered bacterial colonies sensitive to mechanical stress. These results show that host cells actively participate in maintaining bacterial adhesion in the presence of mechanical stress by a plasma membrane dependent process likely involving lipid rafts.

### Lipid microdomain disruption blocks formation of bacteria-induced cellular projections

*N. meningitidis* proliferating on the cellular surface form tight aggregates and trigger a local cellular response with the formation of cellular projections between and around diplococci [18] that could explain the difference of behavior between aggregates in suspension and on the cellular surface. To challenge such a hypothesis, we tested whether cholesterol depletion affected this bacteria-induced cellular response.

**Figure 4:**
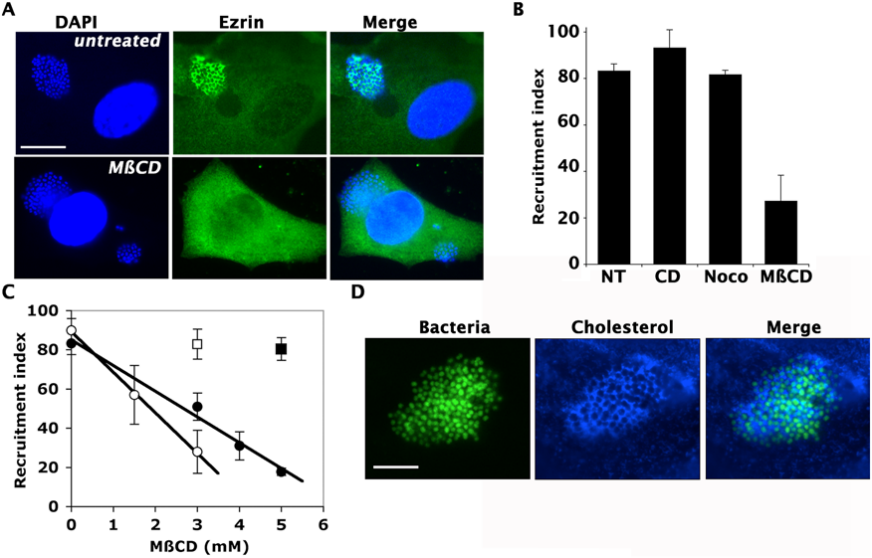
Lipid microdomain disruption by cholesterol depletion prevents bacteria-induced cellular response. The effect of cholesterol depletion by methyl-ß-cyclodextrin (MßCD) on the interaction of *N. meningitidis* with host cells was tested. (A) Ability of *N. meningitidis* microcolonies to recruit cellular components determined by immunofluorescence using Ezrin as a marker. Bacteria and nuclei were stained with DAPI (DAPI); Ezrin was detected with anti-Ezrin polyclonal anti-serum (Ezrin); and images were merged (Merge). Scale bar corresponds to 10 µm. The top set of images are untreated cells and in the bottom set, cells were treated with MßCD. (B) Frequency of bacterial microcolonies efficiently recruiting ezrin (recruitment index) for non-treated cells (NT), in the presence of Cytochalasin D (CD), Nocodazole (Noco), and MßCD. (C) Dose response effect of MßCD with regard to the ability of bacterial microcolonies to reorganize the cellular surface on the surface of epithelial cells (full circles) and on endothelial cells (open circles). To control that the effect of MßCD was due to cholesterol, repletion experiments with added cholesterol were performed with both cell types (squares, open for endothelial cells and full for epithelial cells). (D) Cholesterol localization under bacterial microcolonies. GFP-expressing bacteria were used (Bacteria); Cholesterol was detected with Filipin (Cholesterol); and images were merged (Merge). The scale bar corresponds to 5 µm.

The effect of cholesterol depletion, actin cytoskeleton disorganization and microtubule disruption on the ability of bacterial microcolonies to recruit cellular components in the cortical plaque was first determined by immunofluorescence. Cellular treatment with cytochalasin D or nocodazole did not have any effect on the amount of ezrin recruited by *N meningitidis*, whereas MßCD dramatically decreased the amount of ezrin recruitment (Figure 4A, compare top and bottom panels). Similarly, cytochalasin D or nocodazole did not significantly affect the frequency of microcolonies recruiting ezrin, whereas with MßCD treatment the frequency decreased from 83+/−3% to 27+/−11% (Figure 4B). A dose response curve indicated that the effect of MßCD increased with concentration both on epithelial and endothelial cells (figure 4C, full and open circles respectively). In addition, the effect of MßCD was fully reversed by the addition of cholesterol (Figure 4C, full and open square respectively). Similar results were obtained with other components of the cortical plaque, CD44, ErbB2 or ICAM-1 (not shown). Cholesterol depletion therefore prevents the recruitment of cellular components of the cortical plaque under the bacterial microcolonies.

The potent effect of cholesterol depletion suggested that cholesterol-rich lipid microdomains could be involved in bacteria-induced cellular response. Host plasma membrane cholesterol was detected with Filipin to test this idea. Large amounts of cholesterol were found clustered under 93% of bacterial microcolonies, consistent with a role of lipid microdomains (Figure 4D).

**Figure 5:**
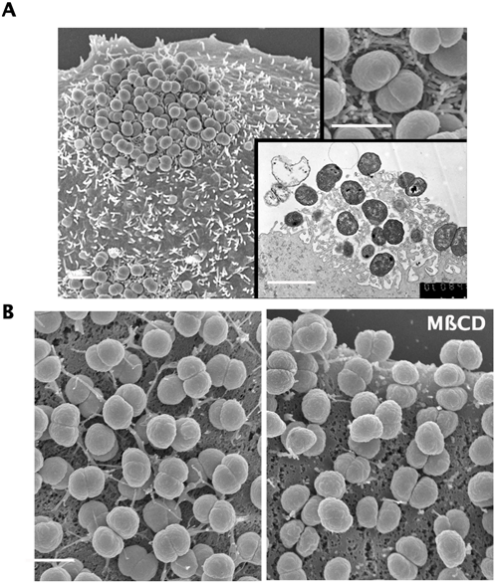
Lipid microdomain disruption prevents the formation of bacteria-induced cellular projections. (A) Human epithelial cells infected with *N. meningitidis* were visualized by electron microscopy to document the organization of bacterial microcolonies in relation with the cellular surface. Low magnification scanning electron microscopy shows bacteria growing in tight aggregates on the cellular surface (scale bars are 1 µm). Higher magnification shows the presence of numerous projections under and around individual bacteria in the aggregates. Transmission electron microscopy analysis of bacterial microcolonies showing the dense network of projections surrounding the bacteria is presented in the lower inset. (B) Scanning electron microscopy analysis of infected endothelial cells shows cellular projections (scale bar is 1 µm). Cells were treated with MßCD during infection and processed for scanning electron microscopy. The number and length of cellular projections is strongly reduced after MßCD (compare left and right panels).

**Figure 6:**
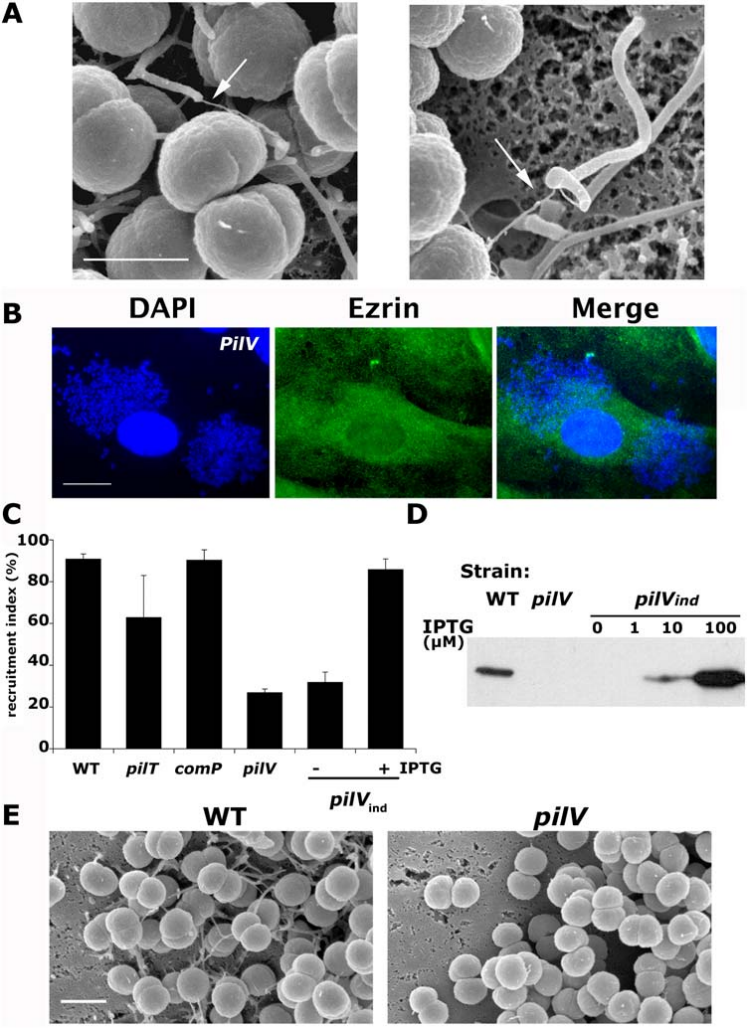
The minor pilin PilV is necessary to induce cellular surface reorganization. Characterization of the ability of a mutant in the *pilV* gene to reorganize host cell plasma membrane of endothelial cells. (A) High resolution scanning electron micrographs showing direct contacts between pili and bacteria-induced cellular protrusions on epithelial cells (arrows, scale bar corresponds to 1 µm). (B) Immunofluorescence analysis of the cellular response to infection with the *pilV* mutant (*pilV*). Bacterial aggregates on the endothelial surface were visualized with DAPI staining (DAPI). Ezrin immunostaining was used as a marker for the recruitment of cytoskeletal components (Ezrin). Scale bar corresponds to 10 µm. (C) Quantification of the effect of the *pilV* mutation on the ability of *N. meningitidis* to recruit ezrin under microcolonies. The frequency of ezrin recruitment under individual microcolonies (recruitment index) is represented for the wild type strain (WT), the *comP*, *pilT*, and *pilV* mutants and the complemented *pilV* strain (*pilV*_ind_) in the presence or absence of inducer (100 µM IPTG). (D) Total protein levels of PilV in the different indicated strains and with different doses of IPTG. (E) Scanning electron microscopy analysis of the cellular surface reorganization induced by wild type (WT) on endothelial cells and absent in the *pilV* mutant (*pilV*), (scale bars correspond to 1 µm).

To document the effect of lipid microdomain disruption on the formation of the filopodia-like protrusions, infected epithelial and endothelial cells were processed for scanning or transmission electron microscopy. Bacteria formed tight aggregates on the epithelial surface and were surrounded by a dense network of fibrous cellular material forming a nest around the bacteria (Figure 5A). Transmission electron microscopy gives a view of the inside of the colony (Figure 5A, lower panel) with projections in tight association with all the bacteria forming the microcolony. Similar images were obtained with bacteria growing on the surface of endothelial cells (Figure 5B) although the network of cellular projections was not as dense as on epithelial cells. Cellular projections could easily be distinguished from type IV pili at higher magnifications although surprisingly few pili fibers could be visualized by scanning electron microscopy in contrast with immunofluorescence studies which visualize a dense meshwork of pili (data not shown and see Figure 6A). Pili visualized on bacterial aggregates by scanning electron microscopy in the absence of cells are not completely smooth, vary in diameter and are rarely observed in bundles larger than 25 nm. In contrast, cellular protrusions on non-infected epithelial cells are smooth, round and homogeneous in diameter, 100 nm. In addition, PFA-fixation of cells prior to infection completely prevents the formation of these structures, indicating that they are of cellular origin. Endothelial cells treated with 5 mM MßCD displayed a dramatic reduction in bacteria-induced cellular projections (Figure 5B, compare left and right panels). Cholesterol repletion allowed the formation of the cellular projections (data not shown). *N. meningitidis*-induced reorganization of the host cell plasma membrane is therefore dependent on the presence of membrane cholesterol for the integrity of lipid rafts, and a strong correlation exists between the presence of bacteria-induced cellular projections and the cohesion of microcolonies.

### Bacteria-induced cellular projection formation depends on minor pilin PilV

**Figure 7:**
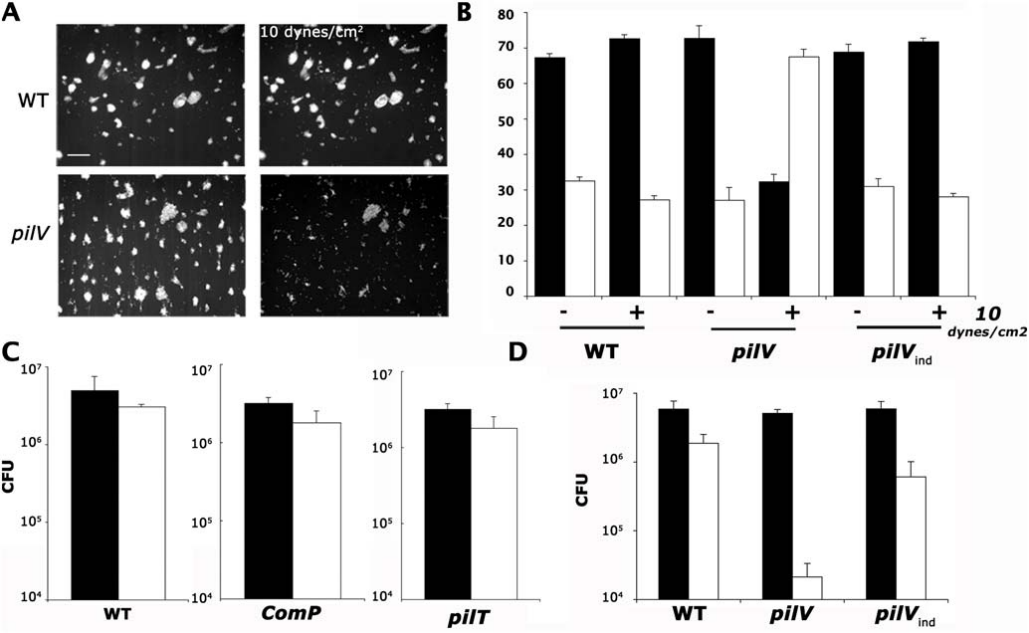
PilV is required to maintain integrity of bacterial microcolonies in the presence of shear stress. The ability of microcolonies formed by a *pilV* mutant to resist shear stress was tested. (A) Microcolonies on a cellular monolayer formed by wild type or the *pilV* mutant expressing GFP were submitted to liquid flow generated force (10 dynes/cm^2^) in a laminar flow chamber. Images of fluorescent bacteria before and after flow increase are presented (scale bars correspond to 50 µm). (B) The morphology of microcolonies was determined before and after shear stress application and the frequency of 2D (white bars) and 3D (black bars) microcolonies determined. (C–D) The number of bacteria adhering to cells before (black bars) and after application of 10 dynes/cm^2^ shear stress (white bars) was determined by the plating assay for the indicated strains.

We next sought to identify bacterial factors involved in triggering the reorganization of the host cell plasma membrane. Previous studies have highlighted the role of type IV pili in triggering *Neisseria gonorrheae*-induced host cell surface reorganization [20]. Deletion of the *pilE* gene encoding the main type IV pilus component leads to a drastic decrease in the clustering of cytoskeletal proteins under *Neisseria gonorrheae* microcolonies. Although this is likely the case in *N. meningitidis*, the situation is complicated by the loss of adhesion of the *N. meningitidis pilE* mutant. Consistent with a direct role of pili, detailed examination of high resolution scanning electron micrographs of infected epithelial cells revealed that in several instances pili were associated with the tip of bacteria-induced filopodia-like structures (Figure 6A). Pilus retraction is important to trigger cortical plaque formation as a *pilT* mutant, unable to retract its pili, is less efficient in triggering this response [20]. In addition to *pilT*, three proteins known as minor pilins, PilX, ComP and PilV are prime candidates as pili components responsible to trigger a cellular response. They are thought to insert in the pilus fiber and could interact directly with the host cell surface to trigger a cellular response [12]–[14]. Because of the strong defect in adhesion of the *pilX* mutant, the potential role of the PilX protein in triggering the cellular response could not be tested. Microcolonies formed by the *comP* mutant recruited ezrin as efficiently as the wild type strain. In contrast with observations in *N. gonorrheae* [14], adhesion efficiency of the *pilV* mutant in *N. meningitidis* was quantitatively indistinguishable from the wild type strain (Figure 7D, black bars). Consistently, the amount of pili present on the surface of the *pilV* mutant was indistinguishable from the wild type strain. This was determined by ELISA on whole bacteria and by immunofluorescence (data not shown). We therefore tested the ability of the *pilV* mutant to recruit ezrin. Under DAPI-stained bacteria in wild type microcolonies growing on endothelial cells, ezrin was abundantly recruited in the typical honeycomb lattice structure. However, the *pilV* mutant was strongly affected in its ability to recruit ezrin (Figure 6B). The same decrease was found for other proteins normally recruited under *N. meningitidis* microcolonies including actin, cortactin, ErbB2, CD44, or ICAM-1 (data not shown). This *pilV* mutant effect was also observed on other eukaryotic cell types (data not shown). To quantitate this effect, the frequency of colonies efficiently recruiting ezrin was determined; while 91+/−2% and 90+/−5% of wild type and *comP* microcolonies efficiently recruited ezrin, only 27+/−2% *pilV* colonies did (Figure 6C, recruitment index). The *pilV* mutation was complemented with an intact copy of the gene. In this construct, the *pilV* gene is regulated at the transcriptional level by an IPTG inducible promoter (*pilV_ind_*). In the presence of 100 µM IPTG, colonies efficiently recruited ezrin with a recruitment index of 86+/−5% (Figure 6C, *pilV_ind,_*). The presence of IPTG led to increased levels of protein as shown by western blot (Figure 6D). Microcolonies formed by the *pilT* strain are morphologically different from those formed by the wild type strain and comparison is difficult. Nevertheless, in the same conditions we found that 63+/−20% of microcolonies formed by the *pilT* strain recruited ezrin (Figure 6C).

To extend these findings, we next evaluated by scanning electron microscopy whether a *pilV* mutant could trigger the formation of cellular projections. Endothelial and epithelial cells were challenged with *N. meningitidis* for 2 hours and cells were processed for scanning electron microscopy. Numerous cellular projections were associated with wild type bacteria growing on endothelial cells (Figure 6E, WT) but this effect was strongly reduced with the *pilV* mutant where bacteria were associated with a flat cellular surface (Figure 6E, *pilV*). Absence of PilV therefore strongly reduces the ability of the bacteria to reorganize the cellular surface and to recruit underlying cellular components.

### PilV dependent cellular projection formation is required to generate shear stress resistant microcolonies

Given that the *pilV* mutant adheres to host cells and forms microcolonies normally but does not induce a cellular response, and that bacterial aggregates in solution in the absence of host cells have low mechanical resistance, we hypothesized the mechanical resistance of the *pilV* mutant microcolonies might also be low. The flow chamber assay was used to test the ability of *pilV* microcolonies to resist to external forces while growing on the epithelial surface. As expected, in the control experiment with wild type bacteria, microcolonies resisted high liquid flow without detectable detachment (Figure 7A, WT). In contrast, microcolonies formed by the *pilV* mutant were disrupted in the presence of 10 dynes/cm^2^ shear stress (Figure 7A, *pilV*). Dynamic observation by video microscopy revealed a gradual disruption of *pilV* mutant microcolonies by mechanical stress (Video S3), in contrast to colonies formed by wild type bacteria where increased shear stress had no visible effect (Video S1). To obtain a quantitative measure of the effect, the morphology of colonies was determined in the presence of flow for the wild type, *pilV* and *pilV*_ind_ strains. As previously shown, the frequency of multilayered wild type microcolonies did not change in the presence of flow (Figure 7B, WT). With the *pilV* strain however, the frequency of multilayered microcolonies decreased from 72+/−3% to 32+/−2% (Figure 7B, *pilV*). Complementation of the *pilV* mutation with a wild type copy restored the ability of the microcolonies to resist mechanical stress (Figure 7B, *pilV_ind_*). PilV is therefore necessary under flow conditions to maintain microcolony cohesion at the cellular surface.

Quantitative measurement of the effect of shear stress was then performed using the plating assay described previously. As expected, increased shear stress had little effect on the wild type or *comP* microcolonies (Figure 7C, WT, *comP*). Colonies formed by the *pilT* mutant were also highly resistant to shear stress. In absence of *pilV* (Figure 7D), bacteria adhered and formed colonies with the same efficiency as the wild type but introduction of shear stress decreased the number of bound bacteria by over one hundred-fold (5.1+/−0.6×10^6^ to 2.1+/−1.2×10^4^). Reintroduction of a wild type copy of the *pilV* gene rescued the phenotype (*pilV*_ind_). The protein PilV is therefore necessary to maintain the integrity of bacterial microcolonies in the presence of mechanical stress. In absence of mechanical stress, however, adhesion and proliferation in aggregates occurs normally.

## Discussion

**Figure 8:**
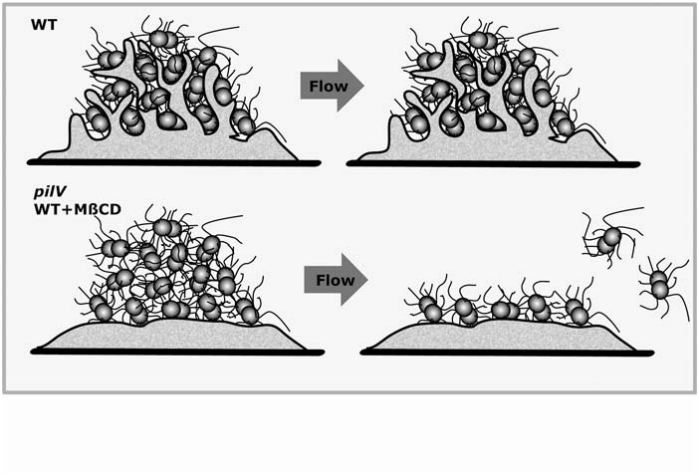
Schematic representation of the link between the ability of microcolonies to resist mechanical force and bacteria induced cellular response. Wild type bacteria trigger a massive reorganization of the plasma membrane and thus resist flow. The *pilV* mutant, in contrast, is able to adhere and form three-dimensional microcolonies but is not able to reorganize the cellular surface and renders the microcolony sensitive to shear stress. A similar effect occurs when cholesterol is depleted from the host cell with MCßD.

*N. meningitidis* can be considered a commensal of the human nasopharynx as these bacteria can survive and multiply in this environment without causing damage, except in the rare but devastating cases when they access the bloodstream. In both cases *N. meningitidis* adopt a specific lifestyle by adhering to the cellular surface where they proliferate in tight, three-dimensional aggregates known as microcolonies in close association with the plasma membrane. We show here that mechanical resistance of microcolonies is dependent on the reorganization of the host cell surface induced by the bacteria. We identify the minor pilin PilV as a central player in inducing this response. Wild type bacteria trigger the formation of cellular projections, which confer ability to resist liquid flow generated mechanical stress (Figure 8, WT). When bacteria are unable to trigger this cellular response, microcolonies become sensitive to shear stress and most bacteria detach except those directly in contact with host cells (Figure 8, *pilV* or MßCD).

Exactly how cellular projections confer mechanical resistance to the microcolony is not completely clear but our results suggest that the formation of cellular projections exert their effect by favoring direct interaction between the bacteria in the aggregate and the host cell plasma membrane. In a bacterial aggregate adhering to the host cell two types of interactions take place: bacteria/bacteria and bacteria/host. Prior to this study the consensus was that three-dimensional microcolony formation was only dependent on the ability of the bacteria to form bacteria/bacteria interactions, a process being dependent on type IV pili. We show here that under conditions mimicking the mechanical stress found in the nasopharynx and the bloodstream, bacterial aggregation is not sufficient to maintain cohesion of the microcolonies. We have previously shown that individual bacteria adhering to the host cell surface are resistant to high shear stress levels [4], indicating the bacteria/cell interaction is shear stress resistant. In the case of wild type bacteria, and in contrast to the *pilV* mutant, every bacterium in the aggregate has the opportunity to directly come into contact with the host cells through the bacteria-induced projections. Reorganization of the host cell plasma membrane induced by the bacteria would therefore stabilize the adhesion of additional bacteria. To describe this collective behavior we propose the term “cooperative adhesion”, to convey the sense that initial adhesion triggers the host cell structural rearrangement that allows subsequent bacteria to adhere in a mechanically resistant microcolony.

An important implication is that the process known as bacterial adhesion is in fact a multistep process. A first consequence of the present findings is that before adhesion, bacteria proliferating in suspension in the bloodstream cannot form aggregates simply because of level of shear stress. In the bloodstream, shear stress levels range between 10 and 100 dynes/cm^2^, and already at 10 dynes/cm^2^ bacterial aggregates disassemble. In the blood, initial attachment therefore likely takes place with individual bacteria or possibly small aggregates. Adhesion starts with the establishment of contact with the host cells by pioneer diplococci followed by proliferation with the formation of aggregates through bacteria-bacteria contacts and concomitantly the induction of cellular projections that maintain direct contact between bacteria and host cells and thus strengthen cohesion. Interestingly certain of these steps can be genetically distinguished, as the *pilV* mutant is only deficient for the step involving the strengthening of microcolony cohesion whereas mutants such as *pilC1* are deficient for the initial attachment step [4].

Previous work showed the importance of *Neisseria spp.* type IV pili in triggering a cellular response. Pilus retraction has been implicated in triggering host cell response as a *pilT* mutant exhibits decreased signaling [20]. From the biophysical point of view it is important to note that the forces created by pilus retraction are in the same range than the forces generated by shear stress if one considers that several pili bundles are involved in contacts with each host cell. According to Goldman *et al.*, under flow-generated shear stress, a rough estimate of the force exerted on a 10 µm wide colony at 100 dynes/cm^2^ would be 30 nN [24], whereas a single pilus bundle can exert forces in the 1 nN range [26]. These observations prompted us to evaluate the mechanical resistance of aggregates formed by the retraction deficient *pilT* strain. We show here that in contrast to the wild type strain, application of shear stress to *pilT* mutant aggregates does not lead to their disruption. This result highlights the unusual properties of *pilT* aggregates and explains why despite a decreased cellular response, *pilT* aggregates growing on a cellular surface are resistant to shear stress. Absence of detachment in the presence of shear stress could also be due to the fact that the effect of the *pilT* mutation on bacteria-induced cellular response is less pronounced than the *pilV* mutation. However, pretreatment of cells with MßCD and infection with the *pilT* mutant led to the formation of shear stress resistant colonies (data not shown) confirming that aggregative properties of the *pilT* mutant are sufficient to explain the absence of detachment upon shear stress application.

Here we show that deletion of a single pilin-like protein PilV severely affects signaling although retraction and twitching motility are not affected in this mutant (not shown and [14]). Pilin-like proteins are thought to insert in the pilus fiber primarily composed of the major pilin PilE. Three such proteins are described in the *Neisseria spp.*, PilX [11], ComP [13] and PilV [14]. Colocalization of the protein with the pilus fiber has been demonstrated directly for PilX by imunogold labeling and electron microscopy and this protein is necessary for bacteria-bacteria aggregation [11],[12]. ComP is necessary for the natural competence of *N. meningitidis* and *N. gonorrhoeae*, probably for the initial step of DNA uptake. In *Neisseria gonorrhoeae*, it was found that the *pilV* mutant is deficient for adhesion although shear stress was not applied [14]. This apparent discrepancy with our results could be due to a difference between bacterial species or to reproducible differences in experimental technique. We found that fixing samples with glutaraldehyde, as was done in the Winter-Larsen *et al.* study, rendered microcolonies formed by the *N. meningitidis pilV* mutant sensitive to washing procedures, suggesting although not proving the two organisms do manifest the same *pilV*-related properties. We found that in the absence of PilV in *N. meningitidis*, type IV pili are unable to trigger efficient reorganization of the host cell membrane. Our results do not formally exclude the possibility that in addition to its defect in inducing a cellular response, the *pilV* mutant also has an aggregation defect revealed by the application of mechanical stress. Further biophysical studies will be needed to address this point. The simplest model to explain the role of PilV in the induction of the cellular response, is that PilV integrates in the pilus fiber outside of the bacterial body and interacts with a receptor on the cellular surface, which in turn stimulates a cellular signaling pathway leading to the reorganization of the cellular surface. Current efforts are focused on demonstrating this direct relationship and identifying a target receptor.

The signaling pathway triggered by type IV pili leading to the formation of cellular protrusions displays distinct properties from the classical pathway for actin-dependent filopodia formation. The actin cytoskeleton has been shown to participate in the pili-dependent process but inhibition of filamentous actin with cytochalasin D or Latrunculin A does not reduce the ability of bacteria to recruit Ezrin or to trigger the formation of cellular projections ([18] and data not shown). Although it is difficult to formally exclude a role for the actin cytoskeleton in the *N. meningitidis*-induced cellular response, our results do suggest that this bacterium takes advantage of unusual cellular pathways to initiate the response. Consistent with its lack of effect on the bacteria-induced cellular projections, cytochalasin D did not render microcolonies sensitive to mechanical stress (data not shown). In contrast, we show here that removing cholesterol from the plasma membrane has a strong effect on the ability of the bacteria to trigger a response and that cholesterol is abundantly recruited below bacterial microcolonies. The recruitment of CD44 [18], src kinase [21] and ezrin under microcolonies also supports a role for lipid microdomains, as these proteins are typically found in these structures. Interestingly, lipid rafts are known to be necessary for a growing list of signal transduction pathways, some of which could be involved in the bacteria-induced process investigated here [25]. Alternatively the effect of cholesterol depletion could be through a modification of the biophysical properties of the membrane. In any case, several pathogens are known to interact with lipid rafts at different stages during the course of pathogenesis [27]. Related to this study, rafts are also important for the initial adhesion of individual bacteria, for instance in the case of the Internalin A-dependent interaction of *Listeria monocytogenes* with host cells [28] or the example of the initial interaction of *Shigella flexneri* with host cells [29]. In other instances, rafts were found to be necessary for invasion of host cells or for intracellular survival [27].

Interaction of *N. meningitidis* with endothelial cells is a new illustration of how bacteria have evolved to exploit host cell function. Intracellular bacteria are known to directly divert cellular pathways to generate a safe niche for the bacteria. In the case of extracellular bacteria such as *N. meningitidis*, adhesion is generally viewed as a passive process reduced to adhesin-receptor interaction [30]. Our results show that even in the case of extracellular bacteria, an intense dialogue takes place with host cells and that the nature of this dialogue influences the properties of the interaction. Interestingly *N. meningitidis* is not the only pathogen to induce the formation of cellular protrusions while proliferating on the cellular surface. The Gram-positive bacterium *Streptococcus pyogenes* (group A Streptococcus) responsible for streptococcal sore throat and invasive soft tissue infection was also found to induce the formation of cellular projections while proliferating at the epithelial surface [16]. Another example is *Bartonella henselae*, a Gram-negative bacillus responsible for the formation of tumors on the skin surface characterized by proliferating endothelial cells associated with clumps of bacteria. Colonization of endothelial cells by aggregates of *B. henselae* is associated with the formation of cellular projections closely resembling those observed with *N. meningitidis* [17]. In contrast to other apicomplexans, the parasite Cryptosporidium resides in an extracytoplasmic parasitophorous vacuole on the cellular surface [31]. After adhesion, long and thick microvilli that cluster around developing trophozoites are analogous to the cellular projections induced by N. meningitidis. Furthermore, host cell membrane reorganization and attachment is also dependent on lipid microdomains [32]. The “pericellular” lifestyle adopted by *N. meningitidis* could be a general process adopted by various pathogens. It will be of interest to test whether certain viruses could also utilize a similar strategy. In any case our results provide a framework for the analysis of the functional significance of pathogen-induced host cell membrane reorganization: these cellular responses provide mechanical resistance to the harsh conditions encountered by extracellular pathogens in their hosts.

From the evolutionary point of view, properties of *N. meningitidis* are positively selected to favor their life in the nasopharynx [33] and certain of these properties then play an important role in the context of pathogenesis. Processes described here can be viewed as the result of such evolutionary pressure. Under pressure to survive in the nasopharynx, *N. meningitidis* has evolved a mechanism to maintain adhesion of microcolonies on the epithelial surface despite harsh mechanical conditions. This adaptive feature becomes a key virulence factor when the bacterium enters the blood and adheres to the cerebral endothelium before invading the brain.

## Materials and Methods

### Reagents and antibodies

Ezrin was detected using selective rabbit polyclonal antisera kindly provided by P. Mangeat (CNRS, Montpellier, France). DAPI (4′,6-diamidino-2-phenylindol) and secondary antibodies directed against rabbit or mouse IgG with Alexa 488 or 564 were purchased from (Molecular Probes, Eugene USA). Mowiol, Methyl-ß-cyclo-dextrin (MßCD) for cholesterol depletion, MßCD-cholesterol for cholesterol repletion, Filipin, Cytochalasin D and Nocodazole were purchased from Sigma (Sigma, Saint Louis, USA).

### Bacterial strains and growth conditions

*N.meningitidis* 8013 clone 12 is a serogroup C clinical isolate, expressing a class I SB pilin, Opa-, Opc-, PilC1+/PilC2+ as described previously [34]. *N. meningitidis* was grown on GCB agar plates (Difco) containing Kellogg's supplements and when required, 100 µg ml^−1^ kanamycin, 4 µg ml^−1^ erythromycin, 50 µg ml^−1^ spectinomycin or 5 µg ml^−1^chloramphenicol at 37°C in moist atmosphere containing 5% CO_2_. *Escherichia coli* transformants were grown on liquid or solid Luria-Bertani medium (Difco) containing 20 µg ml^−1^ chloramphenicol, 50 µg ml^−1^ spectinomycin or 200 µg ml^−1^ erythromycin when necessary. Mutation in the *pilV* gene was introduced into the *N. meningitidis* chromosome by natural transformation of chromosomal DNA extracted from a library of transposition mutants described elsewhere [35]. To complement the *pilV* mutants, the WT *pilV* allele was amplified using primers PilV1 5′-TTAATTAAAGGAGTAATTTTATGAAAAACGTTCAAAAAGGC-3′ and PilV2 5′-GTTTAAACTTAGTCGAAGCCGGGGCAG-3′, which contained overhangs with underlined restriction sites for *PacI* and *PmeI* and cloned in the PCR2.1Topo plasmid. This fragment was restricted with *PacI* and *PmeI* and cloned into *PacI/PmeI*-cut pGCC4 vector, adjacent to lacIOP regulatory sequences [36]. This placed *pilV* under the transcriptional control of an IPTG inducible promoter within a DNA fragment corresponding to an intragenic region of the gonococcal chromosome conserved in *N. meningitidis*. The *pilV*_ind_ allele was then introduced into the chromosome of a *pilV* mutant by homologous recombination. *N. meningitidis* was made to express the green fluorescent protein (GFP) by introducing the pAM239 plasmid by conjugation [4]. To generate the *comP* mutant a portion of the open reading frame was amplified with primers ComP88Fnde 5′-GCATATGTATCGCAATTATGTTGAGAAAG-3′ and 5′-TGGATCCCTACTTAAGTAACTTGCAGTCCTT-3′. This *NdeI* and *BamHI* restricted PCR fragment was cloned into pET14b plasmid (Novagen). The resulting plasmid was restricted with *PmeI*, the blunted spectinomycin cassette [37] was ligated at this site thus interrupting the open reading frame and the resulting plasmid used to introduce the mutation in *N. meningitidis* by transformation. The *pilT* mutant was described elsewhere [38].

### Cell culture

Cells were grown at 37°C in a humidified incubator under 5% CO_2_. Human umbilical vein endothelial cells (HUVECs; PromoCell, Heidelberg, Germany) were used between passages 1 and 8 and grown in Endo-SFM supplemented with 10% heat-inactivated fetal bovine serum (FBS, PAA Laboratories GmbH, Pasching, Austria), 2 mM L-Glutamine (Life-Technologies, Grand Island, USA), 0.5 UI/ml of heparin and 40 µg/ml of endothelial cell growth supplement (Harbor Bioproducts, Norwood, USA). The human endometrial cell line Hec1B (HTB113) was purchased from the American Type Culture Collection (Rockville, Md.) and maintained in DMEM medium supplemented with 10% fetal bovine serum (PAA Laboratories).

### Immunofluorescence

Cells were plated at a density of 10^5^ cells/cm^2^ onto 12-mm diameter glass coverslips coated with fibronectin (10 µg/ml in PBS for 60′). Before the assay, bacteria grown on GCB agar plates were adjusted to OD_600_ = 0.05 and then incubated for 2 hours at 37°C in prewarmed RPMI supplemented with 10% FBS. Approximately 10^7^ bacteria in culture medium were added to 10^5^ cells/well in a 24-well (MOI = 100), allowed to adhere for 30 min, unbound bacteria were washed away and infection was allowed to proceed. At the indicated times, monolayers were processed for immunofluorescence assays. For cytochalasin D, Nocodazole and MßCD treatments, the drug was maintained throughout the entire infection period in culture medium at the indicated concentrations. After infection, cells were fixed with 3.7% paraformaldehyde in PBS for 20 min, permeabilized for 5 min with 0.1% Triton ×100 in PBS and then saturated for 20 min with PBS containing 0.2% gelatin (PBSG) before incubation for 1 hour with the primary antibodies diluted in PBSG. Ezrin anti-serum was diluted 1/1000 (A kind gift from Paul Mangeat, Montpellier, France). Cells were then washed with PBS and incubated for 1 hour in PBSG with anti-rabbit or anti-mouse secondary antibodies conjugated with Alexa 488 (5 µg/ml). DNA was stained with DAPI at a concentration of 100 ng/ml. For cholesterol labeling, Filipin was added after fixation at a concentration of 20 µg/ml. Finally, cells were washed 3 times in PBS and mounted in Mowiol. For quantification, efficiency of recruitment was determined as a recruitment index defined as the percentage of microcolonies efficiently recruiting a given cellular protein. Experiments were performed in triplicate and 50–100 microcolonies were counted for each point.

### Laminar flow chamber experiments

Experiments using the laminar flow chamber were done essentially as described [4]. Endothelial (HUVEC) or epithelial (HEC1b) cells were grown on fibronectin-coated glass slides at a concentration of 10^4^ cells on a 6 mm diameter circular area. Alternatively, disposable flow chambers were used (Ibidi GmbH, München, Germany). Before the assay, bacteria grown on GCB agar plates were adjusted to OD_600_ = 0.02 in prewarmed RPMI medium containing 10% fetal bovine serum and incubated for 90 min at 37°C. GFP expression was induced by adding 1 mM IPTG in the culture medium for an additional 90 min. Cells were infected with 10^6^ bacteria (MOI = 100), adhesion allowed to proceed for 30 min, unbound bacteria washed and infection continued for 2–3 h in an incubator. Infected cells on the glass slide were then placed in a parallel plate flow chamber (3.3 cm×0.6 cm×250 µm, Immunetics, MA, USA) and sealed with vacuum or the Ibidi chamber was placed directly in flow. Medium was maintained at 37°C with a heated platform (Minitub, Germany) and introduced into the chamber using a syringe pump (Vial Medical, Becton-Dickinson or Harvard Apparatus) at various flow rates to produce various wall shear stress levels, using Endo-SFM supplemented with 10% FBS for HUVECs or DMEM supplemented with 10% FBS for Hec1b. Adhesion of bacteria was recorded using an Olympus CKX41 inverted microscope with a 20× objective, equipped with a shutter for the fluorescence lamp and a Hamamatsu ORCA285 CCD camera. The Openlab software (Improvision, UK) controlled the shutter and camera for video time-lapse microscopy. The field under observation corresponded to 425 µm by 320 µm with a resolution of 0.63 µm per pixel. To determine the effect of shear stress on bacterial adhesion a plating assay was performed. Infected cells were detached by Trypsin treatment, collected, serial dilutions performed and a fraction was plated on GCB agar plates.

Estimation of the force exerted on a single bacterium and on a colony adhering to the celluar monolayer in the flow chamber was determined according to Goldman *et al.* [24]. For a static bead next to a wall within the boundary layer, an expression of the force for a static bead next to a wall within the boundary layer is given by F = 1.7005×6πμr^2^γ where μ is viscosity of the fluid, r the bead radius and γ the shear rate.

### Analysis of the mechanical properties of bacterial colonies in absence of cells

Before the assay, bacteria grown on GCB agar plates were adjusted to OD_600_ = 0.05 and then incubated for 2 hours at 37°C in pre-warmed RPMI supplemented with 20% FBS with gentle agitation. The bacterial suspension was concentrated to OD = 0.6 by a 1 min centrifugation at 15000 g followed by resuspension and vortex. Aggregation was then allowed to occur for a period of 30′ in the incubator. For the cone and plate assay, about 150 µl of culture was introduced into a cone and plate rheometer (Brookfield Engineering Laboratories Inc., Middleboro, MA, USA) and submitted to different levels of shear stress (0–10 dynes/cm^2^). Bacteria were then collected and observed microscopically with a 4× lens in a glass bottom 96-well plate (Nunc, Rochester, USA). Aggregate size and number were determined with the Image J software [39]. For the immobilization of bacterial colonies on a glass slide, the 20D9 monoclonal antibody [40] was adsorbed on the glass surface for 60 minutes in PBS at a concentration of 10 µg/ml. The coated glass slide was placed in the flow chamber, bacteria introduced and incubation for periods of 4–6 hours allowed bacterial proliferation. Shear stress was introduced with a syringe pump and images were captured as described above.

### Electron microscopy

For transmission electron microscopy, cells grown on collagen coated, 0.3 mm pore Transwells (Costar) were infected as described above and were fixed overnight at 4°C with a 1∶1 mixture of 2.5% glutaraldehyde and 2.5% paraformaldehyde in cacodylate sucrose buffer (0.1 M cacodylate, 0.1 M sucrose, 5 mM CaCl_2_, 5 mM MgCl_2_, pH 7.2). Monolayers were then stained for 1 hour in a solution of 1% OsO_4_ and placed for 1 hour in 1% uranyl acetate. After dehydration in a graded series of alcohols, cells were embedded with polyester filter in Epon. Thin sections were obtained by using an Ultracut ultramicrotome and analyzed with JEOL-100CX electron microscope.

For scanning electron microscopy, infected cells were fixed in 2.5% glutaraldehyde in 0.1 M cacodylate buffer (pH 7.2) 1 h at room temperature. Samples were washed three times for 5 min in 0.2 M cacodylate buffer (pH 7.2), fixed for 1 h in 1% (wt/vol) osmium tetroxide in 0.2 M cacodylate buffer (pH 7.2), and then rinsed with distilled water. Samples were dehydrated through a graded series of 25, 50, 75 and 95% ethanol solution (5 min each step). Samples were then dehydrated for 10 min in 100% ethanol followed by critical point drying with CO_2_. Dried specimens were sputtered with 10 nm gold palladium, with a GATAN Ion Beam Coater and were examined and photographed with a JEOL JSM 6700F field emission scanning electron microscope operating at 5 Kv. Images were acquired with the upper SE detector (SEI).

### SDS-PAGE, antisera, and immunoblotting

Preparation of protein samples, SDS-PAGE separation, transfer to membranes and immunoblotting were performed using standard biochemistry techniques [41]. To raise antibodies against PilV, we amplified a PCR product corresponding to the *pilV* gene devoid of its N-terminal sequence encoding the first 26 residues of the *Neisseria gonorrheae* PilV preprotein. We used primers PilV forward primer 5′- AGAATTCTACATCCGGCGCGCCCGCCTG -3′ and PilV reverse primer 5′- ATACTGCAGTTAGTCGAAGCCGGGGC-3′, which contained overhangs corresponding to underlined restriction sites for EcoRI and PstI, respectively. The PCR product was first cloned into pCRII-TOPO (Invitrogen, USA) and then excised from pCRII-TOPO with EcoRI and PstI, and subcloned into pMAL-p4x (New England biolabs, USA) already digested by EcoRI and PstI. The final construct contains the coding sequence for maltose binding protein fused upstream and in-frame with the truncated *pilV*. The protein was expressed in *E. coli* BL21::DE3, purified using maltose-agarose, digested with factor X and submitted to ion exchange chromatography. The protein was injected into New Zealand rabbits (Centre Lago, Vonnas, France). For western blot detection, anti-PilV serum was diluted 1/2000 in PBS containing 0.1% Tween20 and 1% milk, secondary antibody HRP-linked anti IgG antibodies was diluted in the same buffer 1/10000. Detection of immobilized antigens was performed by chemiluminescence using ECL Plus detection reagents (Amersham biosciences).

## Supporting Information

Video S1 Video microscopy analysis of the resistance of microcolonies formed by a wild type strain of *N. meningitidis* on endothelial cells. Endothelial cells forming a confluent monolayer on a glass slide were infected with a GFP expressing strain for a period of 3 h and placed in a flow chamber under. Unbound bacteria were removed in the presence of low intensity shear stress (0.04 dynes/cm^2^). Shear stress was increased to 10 dynes/cm^2^. Bacteria forming a microcolony of average size were followed using a time-lapse video microscopy. The video is accelerated 20 times; real time duration is 1 min. The arrow indicates time and direction of application of flow.
(1.06 MB MOV)

Video S2 Analysis of the resistance of microcolonies formed by a wild type strain of *N. meningitidis* on endothelial cells treated with 6 mM MβCD.
(0.87 MB MOV)

Video S3 Effect of shear stress on colonies formed by the *pilV* strain.
(1.13 MB MOV)
